# Generation and Treatment of a Novel Severe Model of Visceral Gaucher Disease by Genetic Therapy

**DOI:** 10.3390/pharmaceutics17050650

**Published:** 2025-05-15

**Authors:** Amy F. Geard, Giulia Massaro, Michael P. Hughes, Patrick Arbuthnot, Simon N. Waddington, Ahad A. Rahim

**Affiliations:** 1 Department of Pharmacology, UCL School of Pharmacy, 29-39 Brunswick Square, London WC1N 1AX, UK; amy.geard.16@ucl.ac.uk (A.F.G.); giulia.massaro.13@ucl.ac.uk (G.M.); michaelhughesp@gmail.com (M.P.H.); 2 Wits/SAMRC Antiviral Gene Therapy Research Unit, Faculty of Health Sciences, University of the Witwatersrand, Johannesburg 2193, South Africa; patrick.arbuthnot@wits.ac.za (P.A.); 3 Maternal & Fetal Medicine, EGA Institute for Women’s Health, Faculty of Population Health Sciences, University College London, London WC1E 6HU, UK; s.waddington@ucl.ac.uk (S.N.W.)

**Keywords:** Gaucher disease, AAV, gene therapy, mouse model, GD Type 1

## Abstract

**Background/Objectives**: Gaucher disease (GD) is an autosomal recessive lysosomal storage disorder caused by mutations in the *GBA1* gene. Type 1 Gaucher disease is characterised by substrate accumulation in the visceral organs, which occurs in combination with acute and chronic neurodegeneration that distinguish type 2 and type 3 GD, respectively. We have previously shown the efficacy of neonatal AAV9 gene therapy for treating type 2 GD and aimed to investigate post-symptomatic administration into a model of type 1 disease. Current murine models of type 1 disease are limited in their recapitulation of early onset phenotypic manifestation and thus we aimed to create a novel model of type 1 in which to test the efficacy of adult gene therapy. **Methods**: The novel AAV-GD1 model was created through intracerebroventricular injection of AAV9 containing the human *GBA1* gene under control of the neuron-specific synapsin promoter (AAV9.hSynI.h*GBA1*) to the pre-existing acute K14-lnl/lnl model of type 2 GD. Administration of AAV9.hSynI.h*GBA1* aimed to restore glucocerebrosidase expression in the brain and extend the lifespan beyond 14 days, allowing the visceral pathology to develop further. The organ pathology was characterised by immunohistochemistry at various time points. Once visceral disease was confirmed, an intravenous injection of AAV9 containing a ubiquitously active CAG promoter driving h*GBA1* (AAV9.CAG.h*GBA1*) was administered to post-symptomatic mice. Animals were aged for 2 and 4 months post-treatment with AAV9.CAG.h*GBA1*, and immunohistochemistry and enzymatic activity were assessed to investigate therapeutic efficacy. **Results**: The AAV-GD1 model displayed visceral pathology in the spleen, lung, and liver from 2 months of age. This allowed us to validate the efficacy of adult gene therapy; intravenous administration of AAV9.CAG.h*GBA1* transiently ameliorated the lung pathology and rescued the spleen pathology up to 4 months post-administration. **Conclusions**: The creation of the novel AAV-GD1 model with more aggressive visceral pathology presents a unique opportunity for investigation of new therapies to treat type 1 GD. AAV9.CAG.h*GBA1* represents a potential therapeutic option for all forms of Gaucher disease.

## 1. Introduction

Gaucher disease (GD) is the most common lysosomal storage disease (LSD) worldwide [1]. It is caused by mutations in the *GBA1* gene that result in absent or reduced activity of the lysosomal enzyme, β-glucocerebrosidase (GCase). GCase is responsible for catalysing the hydrolysis of its substrate, glucosylceramide (GlcCer), to glucose and ceramide [2]. Therefore, the inherited deficiency is characterised by excess accumulation and storage of GlcCer, and, to a lesser extent, glucosylsphingosine (GlcSph) in tissue macrophages. Cells of the macrophage or monocyte lineage are most affected by GCase deficiency, as they are responsible for eliminating erythroid and leukocyte cells, which contain large amounts of glycosphingolipids [2]. Autophagy, mitochondria homeostasis, and the endo-lysosomal pathway are some of the cellular processes that are affected in GD, with engorged macrophages commonly found in the spleen, liver, and bone marrow resulting in key clinical features such as hepatosplenomegaly, bone disease, and cytopenia [3].

Although presenting as a spectrum of pathology, Gaucher disease is broadly classified into three subtypes based on the absence (type 1) or presence, and subsequent severity of CNS involvement (types 2 and 3, known as neuronopathic GD [nGD]). Type 1 GD (MIM 230800) is also referred to as the chronic adult non-neuronopathic form of disease, and it has historically been considered as the form of disease not involving neuropathology. Type 2 (MIM 230900) is the acute neuronopathic form of the disease, and typically presents with the most severe symptoms such as rapid progression and severe neurodegeneration leading to death in infancy or early childhood [4]. Type 3 GD (MIM 321000) is referred to as the juvenile sub-acute neuronopathic form of disease, with visceral symptoms and later-onset of neurological manifestations such as language difficulties, dementia, developmental delays, and slow horizontal saccadic eye movements [5,6]. A continuum of phenotypes has been suggested based on most GD patients demonstrating some form of neurological involvement of varying severity [5,7,8]. Moreover, mutations in the *GBA1* gene have been identified as a common risk factor for Parkinson’s Disease [4,9,10,11].

Enzyme replacement therapy (ERT) is the first line therapy for targeting GD’s visceral pathology. It is administered biweekly via intravenous administration [12] and successfully reduces hepatosplenomegaly, improves thrombocytopenia and anaemia. However, GCase is unable to cross the blood brain barrier (BBB) and therefore cannot be used to treat the neurological manifestations of types 2 and 3 GD. The approach has also shown limited efficacy for treating less-commonly reported GD manifestations such as bone disease [13] pulmonary pathology, lymphadenopathy, and Gaucheroma [14]. Oral substrate reduction therapy (SRT) is an alternative treatment for GD, which is used in patients who cannot tolerate ERT or experience adverse anaphylactic responses to the therapy. It has also been used successfully in combination with ERT in a small cohort of patients [15]. SRT’s mechanism of action inhibits the glucosylceramide synthase enzyme, subsequently reducing the amount of substrate influx into the lysosome and limiting GlcCer accumulation. There are two FDA-approved oral SRT drugs available: Cerdelga^®^ (eliglustat) and Zavesca^®^ (miglustat). Both eliglustat and miglustat have comparable efficacy to ERT but have increased adverse side effect profiles and, despite miglustat being able to cross the BBB, do improve neurologic function in patients [16].

AAV gene therapy has proved to be a safe and effective therapeutic tool for monogenic disorders over the last decade, and the approval of new products such as Zolgensma^®^ (onasemnogene abeparvovec-xioi; Novartis) for Spinal Muscular Atrophy highlights the potential of this therapeutic approach. For neurological disorders, AAV9 is the most commonly used viral vector in both preclinical studies and clinical trials [17]. This is because AAV9 can cross the BBB, allowing use of less invasive routes of administration, and has potential for widespread biodistribution in the brain. AAV vectors also hold great potential for LSDs like GD, where transduced neurons can release AAV-encoded lysosomal enzymes for uptake by adjacent cells. This allows for cross-correction of untransduced cells and enhanced therapeutic efficacy. This is important because small improvements in enzymatic activity may produce clinically meaningful benefits in patients [18]. In combination with the AAV9 capsid, the promoter sequence plays an important role in influencing the cell-specific and strength of transgene expression. The durability of resulting transgene expression in human patients after AAV administration is greater than 10 years [19,20], making it a suitable therapeutic tool for life-long inherited diseases. The current evolving global interest in gene therapy validate the use of viral gene therapy for devastating genetic conditions for which there is currently no cure. Moreover, the number of planned and ongoing clinical trials for Gaucher disease by two independent agencies (NCT05324943 and NCT04411654) highlights the demand for a more effective therapy in this patient population.

Type 1 is the most prevalent form of GD in the western world [21]; however, research into the disease is currently limited by the availability of an animal model that accurately recapitulates phenotypic manifestations with an early age of disease onset. Point mutation models show some evidence of Gaucher cell infiltration in the viscera from 4 months old but no quantitative pathology [22]. Other models, the GD mouse [23] and GBA1 mouse model [24], recapitulate some of the type 1 pathology, however this is only detectable from 5 months old and 14 months old, respectively. This long timeframe for pathology to develop in these models is not conducive to quick investigation of potential therapeutic agents. Furthermore, the models lack quantification of statistically significant pathology. A tamoxifen-induced GD mouse model was created, which has whole body deletion of the *GBA1* gene but can model systemic pathology. Death occurs within 1 week of tamoxifen induction, however, and therefore clinically relevant post-symptomatic gene therapy administration could not be tested [25]. This necessitated the development of a novel model of early onset type 1 GD.

We have previously shown that intravenous AAV9 gene therapy successfully extended the lifespan, improved locomotor function and ameliorated neuropathology of the K14-lnl/lnl mouse model [26,27], an acute model of type 2 neuronopathic GD with a lifespan of approximately 14 days [28]. Successful therapeutic efficacy was observed from intravenous administration in both the fetal [27] and neonatal period [26], with the latter using a neuron-specific promoter. The neonatally administered therapy was effective, however there was limited amelioration of the visceral pathology despite intravenous administration. We therefore investigated a ubiquitously active promoter in an attempt to develop a universal therapy for the Gaucher population, which successfully rescued pre-symptomatic pups when administered intravenously at P0 [29]. These pre-clinical results hold potential for the small portion of type 2 patients where early intervention is possible. Ideally, a universal gene therapy for all forms of GD would be administered early in the neonatal period. However, newborn screening for GD is not a current clinical reality in most countries [30]. Therefore, a model to investigate adult administration in predeveloped type 1 disease was required, mimicking the clinical reality. This would allow us to explore whether gene therapy could reverse pre-existing extensive visceral pathology.

To create a novel model of type 1 GD we employed intracerebroventricular administration of our neuron-directed genetic therapy, AAV9.hSynI.h*GBA1*, to rescue the neurodegeneration of the K14-lnl/lnl model. Previously, this vector had successfully treated the neuropathology in this model when given intravenously [26]. Our brain-directed injection aimed to extend the lifespan of the model beyond 14 days, allowing assessment of the time required for visceral pathology to develop. This model will herein be referred to as the AAV-GD1 model. The AAV-GD1 mice revealed visceral pathology comparable to type 1 disease seen in humans from the early time point of 2 months of age. Thereafter, we used this model to investigate the efficacy of post-symptomatic intravenous administration of AAV9.CAG.h*GBA1* to treat type 1 GD. The ubiquitous CAG promoter used in this vector would ensure expression of therapeutic GBA1 in a broad range of cells in the body.

## 2. Materials and Methods

### 2.1. Plasmid and Viral Vector Production

The cis plasmids *pAAV.hSynI.hGBA1* and *pAAV.CAG.hGBA1* and resulting AAV vectors were produced and tested as described previously [29]. Briefly, the plasmids were cloned into ITR-containing backbones and used to transfect HEK-293 cells. Once increased GCase activity resulting from the plasmids was confirmed, single stranded AAV9 vectors were produced. Endotoxin-free plasmid preparations were performed and provided to Vector Biolabs. The AAV vectors were produced by standard triple transfection of HEK293 cells and purified by ultracentrifugation of density gradients and subsequently caesium chloride. Finally, the vectors were diluted in phosphate buffered saline (PBS) to a final concentration of 6 × 10^13^ vg/mL.

### 2.2. Animal Colony Maintenance and Viral Vector Administration

All in vivo investigations were performed using the K14-lnl/lnl mouse model, and the newly developed AAV-GD1 model described. Mouse procedures and welfare were approved by the University College London Animal Welfare and Ethical Review Board (AWERB) in accordance with the project and personal licences granted by the UK Home Office and the Animal (Scientific Procedures) Act of 1986. The Animal Research Reporting of In Vivo Experiments (ARRIVE) guidelines were also closely followed. The animals were kept on a 12-h light/dark cycle with unlimited access to food and water. Mice were housed in individually ventilated cages (IVCs), with appropriate husbandry and environmental enrichment. The breeding colony was maintained as heterozygotes and genotyped as previously described [28]. The mice were regularly monitored and culled if the humane end point was reached. This was defined as greater than 15% body weight loss, or if the mouse presented with spasticity, paralysis, neck hypertension, or unconsciousness for more than 4 h. The AAV-GD1 model was created through intracerebroventricular administration of 7.2 × 10^11^ vg of AAV9.hSynI.h*GBA1* vector to postnatal day 1 (P1) K14-lnl/lnl knockout (KO) pups. An amount of 5 μLof the vector was administered bilaterally using a Hamilton syringe (Hamilton company, Reno, NV, USA) and the injection site was identified as previously described [31]. The mice were aged to either 2 or 4 months old. To investigate the efficacy of AAV9.CAG.h*GBA1* at reversing or ameliorating the pathology in an adult type 1 model of GD, AAV-GD1 animals were injected intravenously (IV) with 4.6 × 10^14^ vg/kg at 2 months of age. Treated animals were then aged to 2 or 4 months post-IV injection. AAV-GD1 mice were randomly allocated to each experimental group and no animals or data points were excluded in the study. Researchers were unblinded to the experimental groups during the study. We calculated the group sizes (*n* = 3) based upon immunohistochemical quantifications performed in previous studies using GUSB [27], Synapsin [26] and CAG promoters, using the probability of finding an effect set at 80% and the probability of incorrectly rejecting the null hypothesis when it is true set at 0.05 (alpha). We used the Power and Sample Size calculator [32].

### 2.3. Sample Collection

Mice were collected at the experimental end point by transcardial prefusion with phosphate buffered saline (PBS, Thermo Fisher Scientific, Massachusetts, USA) under terminal isoflurane anaesthesia. Cardiac puncture was performed at the time of collection to assess enzymatic activity in the plasma. Blood was collected in EDTA tubes (Sarstedt) and centrifuged at 2000× *g* for 15 min. Plasma was transferred to a new tube and stored at −80 °C for future use. End-stage plasma readings were used as sufficient volumes of plasma could not be collected during the in-life portion of the study in smaller animals. The organs harvested included the brain, lungs, liver, and spleen. One part of each organ was fixed in 4% paraformaldehyde (PFA) solution for 48 h at 4 °C, before transfer to a cryopreserving solution of 30% sucrose, again at 4 °C. The other half of the organ was snap frozen on dry ice and stored at −80 °C for enzymatic analysis.

### 2.4. Immunohistochemical and Histological Analysis

Fixed tissues were cryosectioned at 40 μm thickness using a Leica CM3050S cryostat (Leica Microsystems, Wetzlar, Germany) at a constant temperature of −20 °C. Immunohistochemical staining was used to assess neuroinflammation and lysosomal status in the brain, as well as inflammation in the key visceral organs. Staining was performed as described previously [26], with brain sections stained with primary antibodies for CD68 (1:2000, MCA1957 Bio-Rad, Hercules, CA, USA) and GFAP (1:1000, MAB3402 Millipore, St. Louis, MO, USA) to assess microglial activation and astrocyte activation, respectively, LAMP1 (1:2000, ab24170 Abcam, Cambridge, UK) to investigate lysosomal accumulation, and GCase (1:1000, G4171 Sigma-Aldrich, Milwaukee, WI, USA) for both endogenous and vector-mediated GCase protein expression. Biotinylated anti-rat (BA-9400, Vector Laboratories, Newark, NJ, USA) or anti-rabbit (BA-1000, Vector Laboratories, Newark, NJ, USA) secondary antibodies were used at a final concentration of 1:1000, diluted in 0.3% Triton X-100 TBS (TBS-T). Visceral organs were also assessed for macrophage and lysosome accumulation using CD68 and LAMP1, respectively. Hematoxylin and Eosin (H&E) staining of visceral organs was also performed to assess tissue architecture and Gaucher cell accumulation. To achieve this, sections were mounted onto chrome-gelatine coated slides to air dry. The dried sections were protected from the light and stained with filtered 0.1% Mayer Hematoxylin (Sigma-Aldrich) for 10 min. The sections were rinsed in distilled water and stained with 0.5% Eosin solution (Sigma-Aldrich). The stained sections were rinsed again until excess Eosin staining was removed and subsequently dehydrated in increasing concentrations of ethanol (50%, 70%, 95%, 100%). Finally, the slides were incubated in Histoclear (Geneflow, Lichfield, CT, USA) for 30 min and cover slipped using a DPX mountant for histology (Sigma-Aldrich, Milwaukee, WI, USA).

### 2.5. Microscope Imaging and Quantitative Analysis

Average immunoreactivity of GFAP, CD68, LAMP1, and GCase antibodies was measured by quantitative thresholding image analysis as previously described [33]. Briefly, 10 non-overlapping images of discrete regions were captured using a Nikon DS-Fi1 camera (Nikon, Tokyo, Japan) attached to a Nikon Eclipse E600 microscope. Light intensity and microscope calibration settings were kept constant throughout imaging of the brain and visceral organ sections stained with the same antibody. The level of immunoperoxidase-based staining was quantified using the Image-Pro Premier Analysis System (Media Cybernetics, Rockville, MD, USA). A minimum threshold value for the detection of foreground pixel intensity above the background was assigned for a specific organ group stained with a certain antibody. The level of quantified staining was defined as pixel intensity above the assigned threshold, which was expressed as a stained percentage of the total area of the image which was measured.

### 2.6. Enzymatic Activity Assay

GCase enzymatic activity was measured as described previously [27]. Briefly, samples were prepared by homogenising approximately 1 g of frozen tissue in distilled water on ice, centrifugation at 12,000× *g* for 20 min at 4 °C and recovery of the supernatant. Protein concentration was determined using a Pierce BCA Assay (Thermo Fisher Scientific, Waltham, MA, USA). Samples were then incubated with the well-established synthetic substrate 4-methylumbelliferone-β-glucopyranoside (4-MU-βGlu, Sigma-Aldrich, Milwaukee, USA) in 0.15 M extraction buffer (pH 5.9) at 37 °C for one hour. The reaction was stopped by the addition of 1M glycine buffer (pH 11) and resulting fluorescence levels were measured using the FluoStar Optima Plate Reader (excitation wavelength: 360 nm, emission wavelength: 450 nm). GCase enzymatic activity was expressed as nmol/hr/μg.

## 3. Results

### 3.1. AAV-GD1 Mice Survive to at Least 6 Months of Age and Develop Splenomegaly from 2 Months Old with No Evidence of Neuropathology

**Figure 1:**
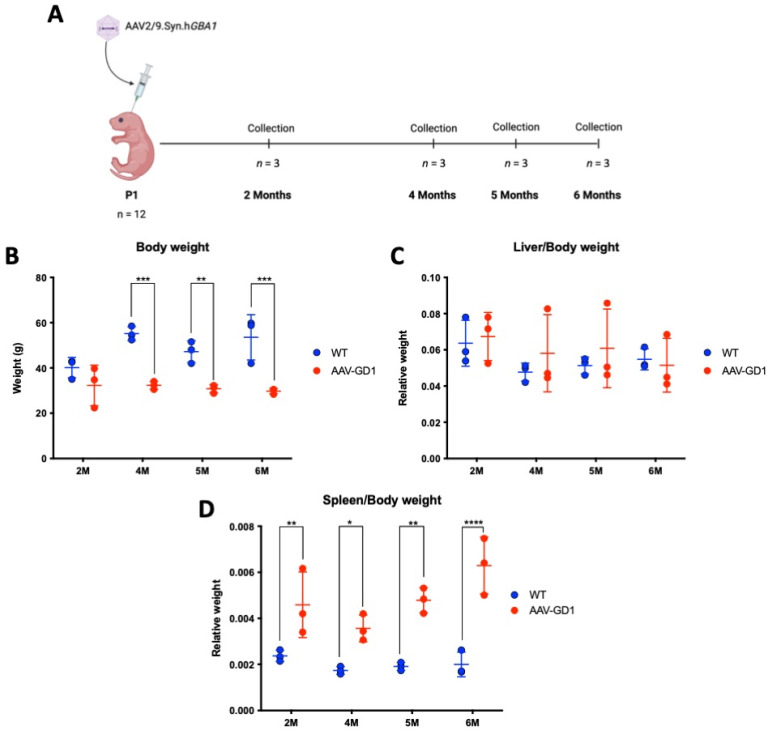
Experimental procedure and weights of AAV-GD1 mice compared to wild-type controls. (**A**) Experimental procedure to create and characterise the AAV-GD1 model using ICV administration of AAV9.hSynI.h*GBA1* to P1 K14-lnl/lnl KO pups. Once survival beyond the 14-day critical threshold was achieved, mice were sacrificed at 2, 4, 5, and 6 months of age (*n* = 3). (**B**) End-stage body weight of AAV-GD1 animals compared to WT controls (*n* = 3); (**C**) liver weights expressed as a portion of total body weight; and (**D**) spleen weight expressed as a portion of total body weight indicated splenomegaly development from 2 months old. (Data presented as average ± SD. Statistical analysis: two-way ANOVA, adjusted for multiple comparisons using Sidak’s multiple comparison test. * *p* < 0.05; ** *p* < 0.01; *** *p* < 0.001; **** *p* < 0.0001).

K14-lnl/lnl KO mice received 7.2 × 10^11^ vg of AAV9.hSynI.h*GBA1* via intracerebroventricular (ICV) injections at postnatal day 1 (P1). This created the AAV-GD1 model that significantly extended the lifespan compared to the 14-day lifespan of untreated knock-out mice. The AAV-GD1 animals were sacrificed at 2, 4, 5, and 6 months of age and compared to age-matched wild-type (WT) controls (*n* = 3 per group) (Figure 1A). No significant change in the weight of AAV-GD1 mice was observed at 2 months of age. However, AAV-GD1 mice were significantly smaller at 4, 5, and 6 months of age (Figure 1B). To investigate hepatosplenomegaly, a characteristic feature of GD in humans, the liver and spleen weights were measured at the time of sacrifice. These were normalised to the body weight to account for any difference in the size of the animals. No significant difference was observed for the liver–body weight ratio (Figure 1C), while the spleen–body weight ratio was significantly increased for AAV-GD1 mice at all ages (Figure 1D). This indicated that splenomegaly, a key characteristic of Type 1 Gaucher disease, had developed in this model by 2 months of age.

**Figure 2:**
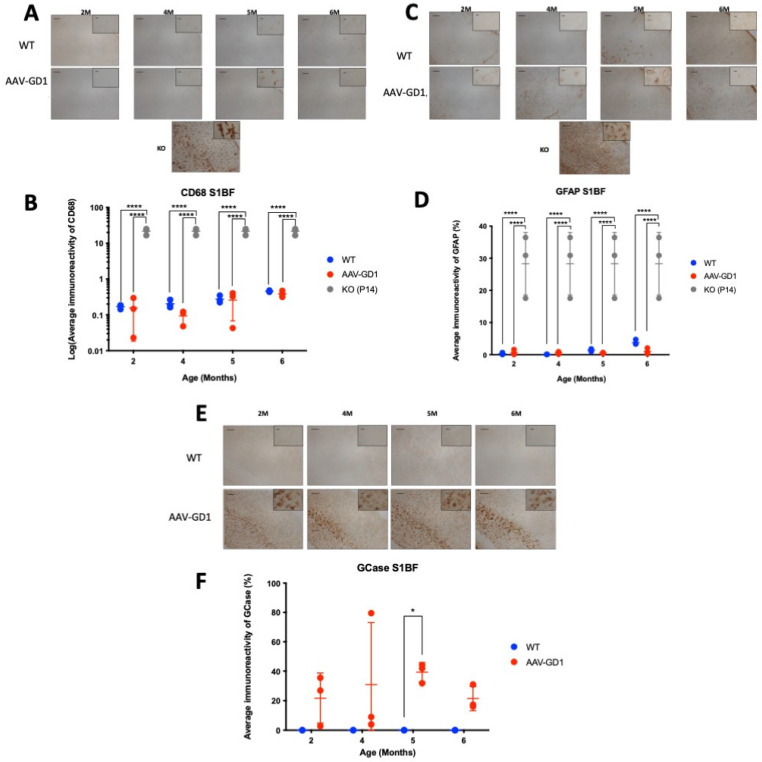
Ameliorated neuropathology and continued GCase expression in AAV-GD1 mice post-ICV administration. (**A**) Representative images of CD68 staining in the S1BF region of the brain from WT and AAV-GD1 mice, with a P14 KO brain included as a positive control, to assess microglial activation. (**B**) Quantification of CD68 immunoreactivity revealed significantly reduced microglial activation in AAV-GD1 animals at all ages compared to untreated P14 KO controls. (**C**) Representative images of astrogliosis are indicated by GFAP immunostaining in the S1BF and (**D**) quantification of GFAP immunoreactivity, which was also significantly reduced in AAV-GD1 animals compared to KO mice. (**E**) Light microscopy images of the S1BF stained for glucocerebrosidase and (**F**) quantification of immunostaining revealed persistent GCase expression even 6 months post-ICV AAV9.hSynI.h*GBA1* administration. Scale bar: 100 µm, higher magnification insert: 65 µm. *N* = 3 per cohort. (Data presented as average ± SD. Statistical analysis: two-way ANOVA, adjusted for multiple comparisons using Sidak’s multiple comparison test. * *p* < 0.05; **** *p* < 0.0001).

The K14-lnl/lnl type 2 mouse model typically presents with acute and extensive neuroinflammation, microglial activation, and astrogliosis at the end stage [26,27,28,29], which is not typical of type 1 disease. We examined the somatosensory barrel (S1BF) region of the cortex as it is one of the most affected regions in the mouse model and is representative of the human course of disease. We compared AAV-GD1 animals to age-matched wild-type controls, with postnatal day 14 (P14) KO brains included as a positive control for neuropathology. The brains of untreated K14-lnl/lnl knockout mice typically show extensive microglia proliferation. This was significantly ameliorated in the cortex of brains of AAV-GD1 mice at all ages (*p* < 0.0001) and restored to wild-type levels (Figure 2A,B). Activated astrocytes also serve as an indicator of neuroinflammation, which is detected using the GFAP marker. These results mirrored those seen for CD68. The level of astrocyte activation in AAV-GD1 animals was restored to the WT level, significantly less than in untreated K14-lnl/lnl KO mice (*p* < 0.0001) (Figure 2C,D). This is expected for a model of type 1 Gaucher disease where neuropathology would not be a defining feature. Additionally, we investigated expression of glucocerebrosidase post-ICV administration, which showed long-lasting, widespread overexpression of glucocerebrosidase in AAV-GD1 brains even at 6 months post-injection (Figure 2E,F).

### 3.2. Engorged Macrophages and Gaucher Cells Detected from 2 Months Old in AAV-GD1 Mice

**Figure 3:**
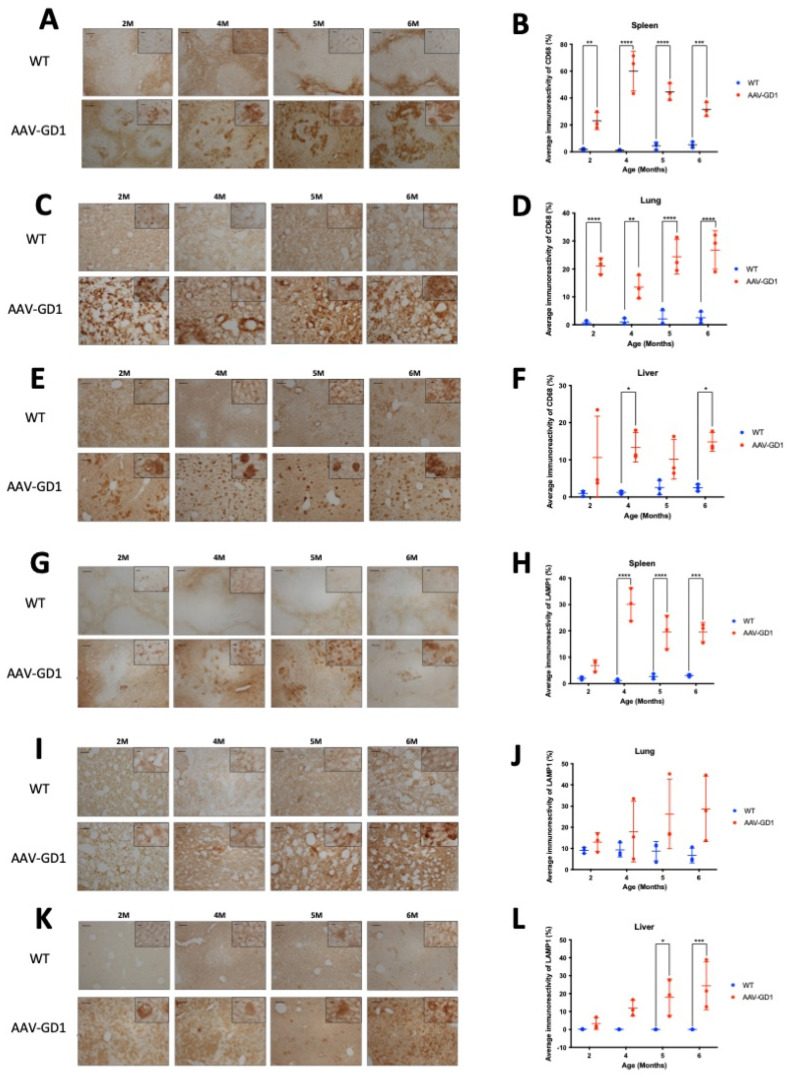
Visceral pathology is evident from 2 months old in AAV-GD1 mice. Representative light microscopy images and relevant quantification of macrophage activation by CD68 staining in the (**A**,**B**) spleen, (**C**,**D**) lung, and (**E**,**F**) liver of AAV-GD1 mice compared to age-matched wild-type controls. LAMP1 immunostaining and quantification was also performed to assess lysosomal accumulation in the (**G**,**H**) spleen, (**I**,**J**) lung, and (**K**,**L**) liver of WT and AAV-GD1 animals. These results suggest that some pathology is already evident in AAV-GD1 animals by 2 months of age. Scale bar: 100 µm, higher magnification insert: 65 µm. *N* = 3 per cohort. (Data presented as average ± SD. Statistical analysis: two-way ANOVA, adjusted for multiple comparisons using Sidak’s multiple comparison test. * *p* < 0.05; ** *p* < 0.01; *** *p* < 0.001; **** *p* < 0.0001).

Type 1 GD visceral organ disease is most commonly observed in the spleen and liver, with some lung pathology. We aimed to determine the extent of visceral pathology in the AAV-GD1 mouse, and the time of disease onset. Immunostaining for CD68 demonstrated engorged macrophages in all tissues which were more abundant than in WT animals. Significant macrophage activation was observed in the spleens of AAV-GD1 mice (Figure 3A) at 2 months (*p* = 0.0045), 4 months (*p* < 0.0001), 5 months (*p* < 0.0001), and 6 months of age (*p* = 0.0006) (Figure 3B). The engorged macrophages were largely observed in the white pulp (Figure 3A). Significant accumulation of engorged macrophages was also seen in the lungs of AAV-GD1 mice (Figure 3C) at 2 months (*p* < 0.0001), 4 months (*p* = 0.0058), 5 months (*p* < 0.0001), and 6 months (*p* < 0.0001) (Figure 3D). Higher magnification images revealed engorged macrophages in the lungs of AAV-GD1 mice in comparison to the smaller punctate staining characteristic of macrophages seen in healthy wild-type tissue (Figure 3C). Engorged macrophages were also observed in the liver (Figure 3E), which were significantly increased in AAV-GD1 mice at 4 months (*p* = 0.0258) and 6 months of age (*p* = 0.0229) (Figure 3F). Although analysis at the other timepoints did not show significant differences, both 2-month (*p* = 0.0926)- and 5-month (*p* = 0.2416)-old AAV-GD1 animals displayed a higher prevalence of engorged macrophages (Figure 3E). This increase, although not significant, was greater and more consistent in the 5-month-old AAV-GD1 mice than in 2-month-old animals.

Lysosomal-associated membrane protein 1 (LAMP1) immunostaining was used to detect enlarged lysosomes in the visceral organs. Lysosomes become enlarged in Gaucher disease due to the accumulation of glycosphingolipids. Light microscopy of the spleen revealed enlarged lysosomes in the white pulp of AAV-GD1 animals at all ages (Figure 3G). Quantification showed a significant increase in the average immunoreactivity at 4 months (*p* < 0.0001), 5 months (*p* < 0.0001), and 6 months of age (*p* = 0.0001) (Figure 3H). In the lung, quantification of immunostaining revealed no significant increase in enlarged lysosomes at 2 months (*p* = 0.9823), 4 months (*p* = 0.7587), 5 months (*p* = 0.1681), or 6 months of age (*p* = 0.0572) in AAV-GD1 mice (Figure 3J). However, immunostaining tended to be higher in all AAV-GD1 animals, and higher magnification imaging revealed the presence of enlarged lysosomes in the AAV-GD1 cohort, particularly at 5 and 6 months of age. This was distinct from the small punctate lysosomal staining observed in the wild-type tissues (Figure 3I). The inability of the quantification software to detect lysosome-specific immunostaining relative to the elevated background staining level may explain why no statistically significant results were obtained, despite the obvious presence of enlarged lysosomes in the lungs of AAV-GD1 mice. Similar to the lung, the liver tissue from all AAV-GD1 mice displayed an increase in enlarged lysosomes (Figure 3K). This was statistically significantly increased at 5 months (*p* = 0.0110) and 6 months of age (*p* = 0.0008). The increase was not statistically significant at 2 months (*p* = 0.9598) and 4 months old (*p* = 0.1341) (Figure 3L); however, enlarged lysosomes were visible under both low- and high-magnification microscopy. Moreover, the presence of enlarged lysosomes seemed to increase with age in the AAV-GD1 cohort.

**Figure 4:**
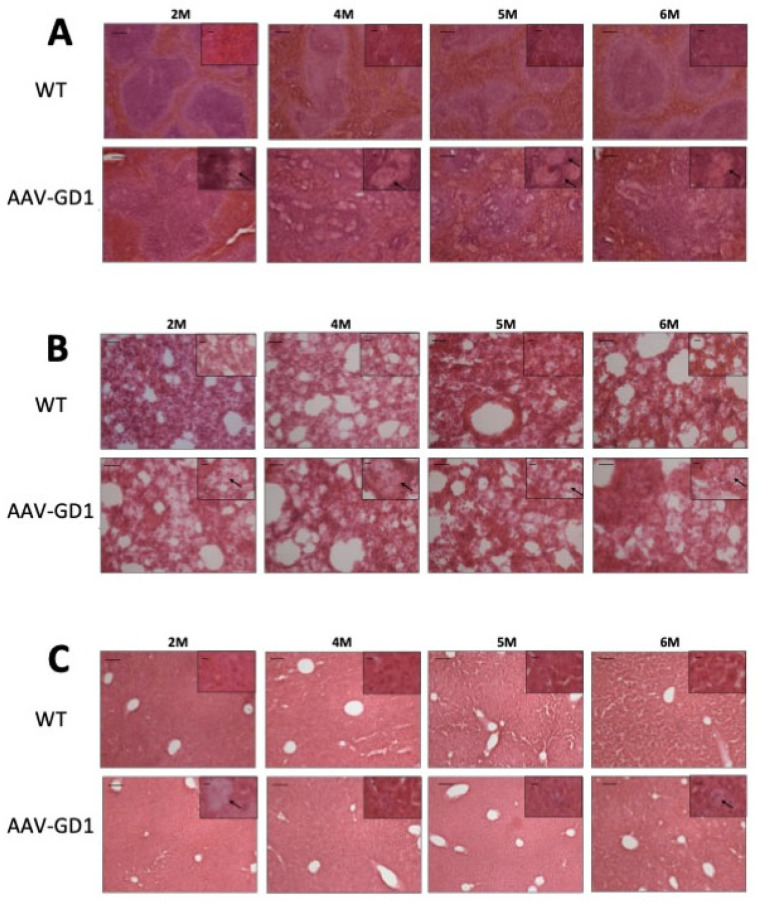
Gaucher cell infiltration and disrupted tissue architecture in the viscera of AAV-GD1 mice. H&E staining of (**A**) spleen, (**B**) lung, and (**C**) liver tissue from AAV-GD1 mice and age-matched controls revealed infiltrating Gaucher cells (black arrow) and disrupted tissue architecture, particularly the white pulp region of the spleen, from 2 months of age. Scale bar: 100 µm, higher magnification insert: 65 µm. *N* = 3 per cohort.

Tissue architecture was also assessed using microscopy analysis of H&E-stained tissues. The overall structure of lung and liver samples from AAV-GD1 animals appeared similar to wild-type tissues. However, enlarged Gaucher cells were observed in the lung, liver, and spleen tissues from 2 months old (Figure 4A–C), confirming the results observed with CD68 staining. Spleen architecture in tissue from AAV-GD1 mice was significantly disrupted with enlarged Gaucher cells, particularly in the white pulp region from 2 months of age (Figure 4A). This was also evident in spleens of AAV-GD1 animals that were stained for CD68 and LAMP1 markers.

### 3.3. Post-Symptomatic Genetic Therapy Rescues Splenic Pathology in AAV-GD1 Model with Limited Efficacy in Other Organs

**Figure 5:**
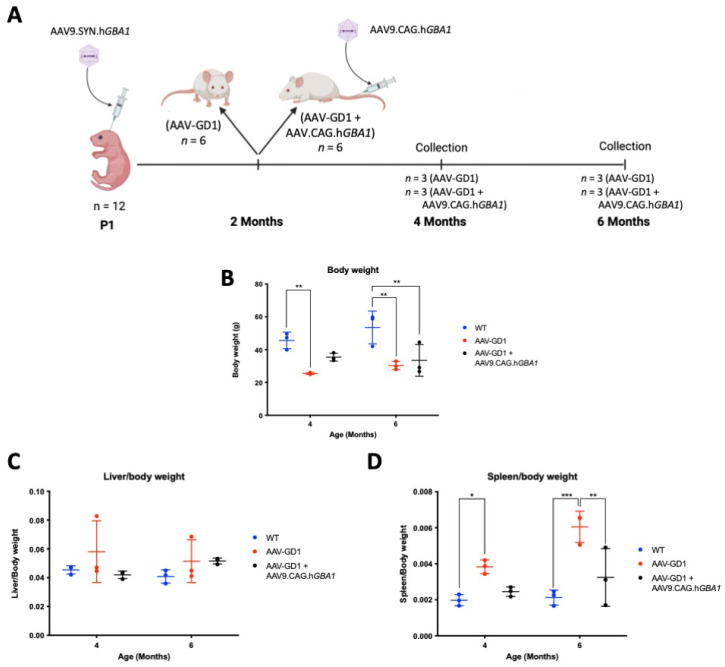
Experimental procedure and efficacy of post-symptomatic intravenous AAV gene therapy in treating body weight and hepatosplenomegaly of AAV-GD1 model. (**A**) AAV-GD1 mice were created as described previously (*n* = 12), and at 2 months old half of the AAV-GD1 groups received an intravenous AAV9.CAG.h*GBA1* injection. Two months post-IV administration (i.e., 4 months old), half of the AAV-GD1 (*n* = 3) and AAV-GD1 + AAV9.CAG.h*GBA1* (*n* = 3) experimental groups were collected. The remaining mice (*n* = 3 per cohort) were aged for a further 2 months and collected 4 months post-IV administration (i.e., at 6 months old). (**B**) End-stage body weight of treated AAV-GD1 animals compared to age-matched wild-type and untreated controls. (**C**) Liver weights expressed as a portion of total body weight and (**D**) spleen weight expressed as a portion of total body weight revealed splenomegaly was improved in treated AAV-GD1 animals at 6 months old. (Data presented as average ± SD. Statistical analysis: two-way ANOVA, adjusted for multiple comparisons using Sidak’s multiple comparison test. * *p* < 0.05; ** *p* < 0.01; *** *p* < 0.001).

Following development and characterisation of the AAV-GD1 model, we determined that sufficient pathology was present at 2 months old to mimic type 1 GD. We therefore selected this time as the point of intervention to assess the efficacy of post-symptomatic intravenous AAV9.CAG.h*GBA1* administration for treating type 1 GD. Two-month-old AAV-GD1 mice were treated with an intravenous administration of 4.6 × 10^14^ vg/kg AAV9.CAG.h*GBA1* and then sacrificed at 2 or 4 months post-intravenous injection (*n* = 3 per time point), along with age-matched wild-type and untreated AAV-GD1 mice (Figure 5A). Untreated AAV-GD1 animals were smaller than wild-type controls at 4 months old (*p* = 0.0056), while untreated (*p* = 0.0019) and treated AAV-GD1 (*p* = 0.0056) animals were smaller than wild types at 6 months old (Figure 5B). As observed previously, no significant hepatomegaly was detected in either treated or untreated AAV-GD1 mice group at 4 and 6 months of age (Figure 5C). Splenomegaly was rescued in treated AAV-GD1 mice, with statistically significant improvements observed in 6-month-old animals (*p* = 0.003) (Figure 5D). This indicated a significant reversal of splenomegaly in this model.

**Figure 6:**
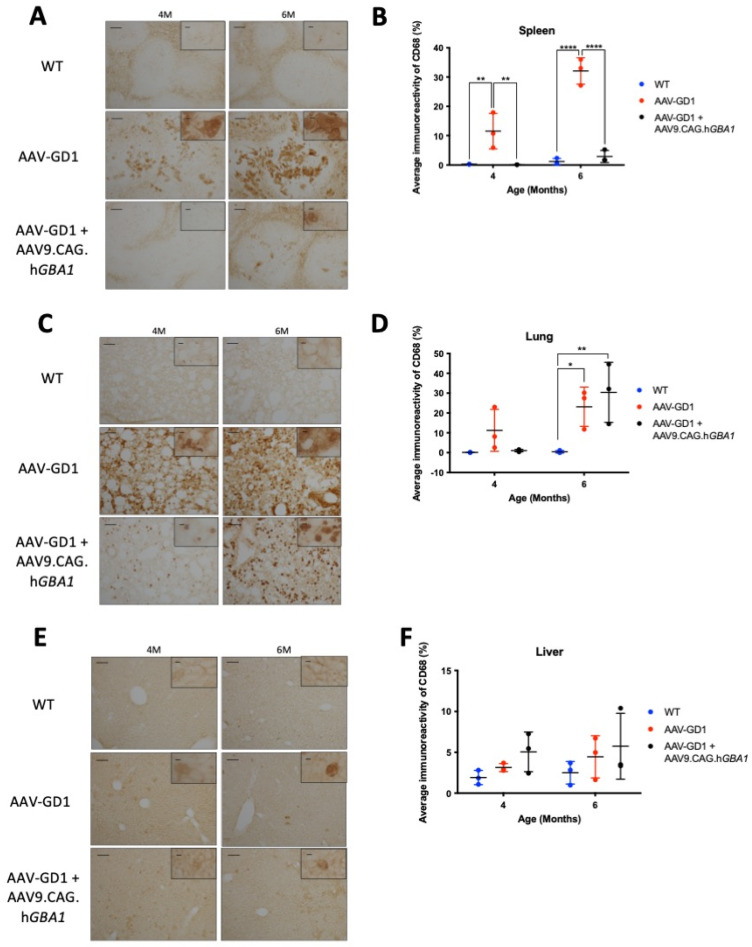
Post-symptomatic intravenous AAV9.CAG.h*GBA1* partly ameliorates visceral pathology. Representative light microscopy images and relevant quantification of macrophage activation by CD68 staining in the (**A**,**B**) spleen, (**C**,**D**) lung, and (**E**,**F**) liver of untreated and treated AAV-GD1 mice compared to age-matched wild-type controls. These results suggest that intravenous gene therapy successfully treats some organs, such as the spleen, while others such as the lung remain refractory to treatment. Scale bar: 100 µm, higher magnification insert: 65 µm. *N* = 3 per cohort. (Data presented as average ± SD. Statistical analysis: two-way ANOVA, adjusted for multiple comparisons using Sidak’s multiple comparison test. * *p* < 0.05; ** *p* < 0.01; **** *p* < 0.0001).

Macrophage activation in the spleens of treated animals was significantly reduced at both 4 months (*p* = 0.0028) and 6 months old (*p* < 0.0001) (Figure 6B). Light microscopy revealed that inflammation in AAV-GD1 spleens seemed to escalate with increasing age, as indicated by a higher prevalence of large, engorged macrophages in the white pulp of spleen tissue at 6 months old. These large macrophages were largely absent in treated AAV-GD1 animals with the exception of some solitary engorged macrophages seen in the white pulp region of 6-month-old animals (Figure 6A). In the lung, no significant inflammation was detected by immunostaining in 4-month-old untreated or treated AAV-GD1 mice (Figure 6D). Light microscopy images of lung sections from 4-month-old untreated AAV-GD1 mice revealed a higher prevalence of engorged macrophages compared to those seen in the treated animals which displayed smaller, more punctate staining (Figure 6C). At 6 months old, both the untreated and treated AAV-GD1 mice showed a significant increase in average immunoreactivity compared to the wild-type control (*p* = 0.0208 and *p* = 0.0032, respectively). Light microscopy of lung tissue from 4- and 6-month-old treated AAV-GD1 mice revealed that the size and number of engorged macrophages had increased between 4 and 6 months (Figure 6C). In the liver, macrophage activation was not observed to the same extent as previously observed in AAV-GD1 animals. No significant increase was observed at 4 or 6 months when comparing untreated (*p* = 0.8952 or *p* = 0.6835, respectively) or treated AAV-GD1 mice (*p* = 0.3188 or *p* = 0.2929, respectively) (Figure 6F). The treated AAV-GD1 mice tended to have an increased average immunoreactivity at both 4 and 6 months of age. However, light microscopy revealed the macrophages to be smaller, more diffuse markings compared to the darker, more engorged macrophages seen in the AAV-GD1 mice, particularly at 6 months (Figure 6E).

**Figure 7:**
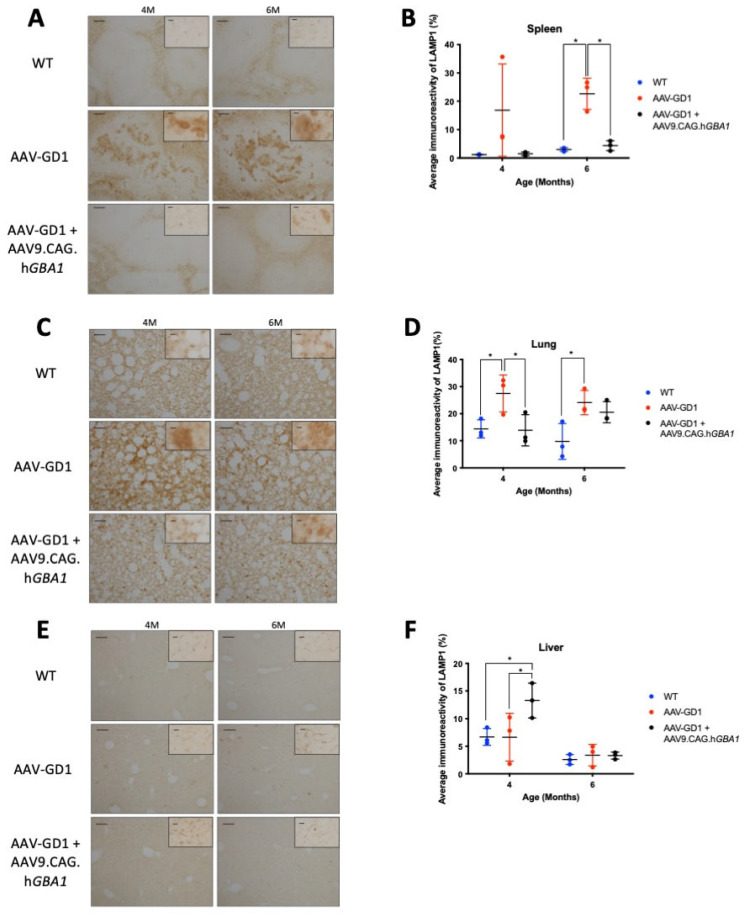
Post-symptomatic intravenous AAV9.CAG.h*GBA1* partly ameliorates visceral lysosomal accumulation. Representative light microscopy images and relevant quantification of LAMP1 immunostaining to assess lysosomal accumulation in the (**A**,**B**) spleen, (**C**,**D**) lung, and (**E**,**F**) liver of WT and AAV-GD1 mice compared with those AAV-GD1 animals treated with AAV9.CAG.h*GBA1*. Scale bar: 100 µm, higher magnification insert: 65 µm. *N* = 3 per cohort. (Data presented as average ± SD. Statistical analysis: two-way ANOVA, adjusted for multiple comparisons using Sidak’s multiple comparison test. * *p* < 0.05).

Lysosomal accumulation was also visualised using LAMP1 immunostaining of the spleen, lungs, and liver. Lysosomal accumulation in the spleen was rescued in the 6-month-old AAV-GD1 treated animals (*p* = 0.0238) (Figure 7B). Light microscopy revealed enlarged lysosomes in the white pulp region of untreated AAV-GD1 animals at 4 and 6 months old, which was largely absent in treated animals at the same time points (Figure 7A). In the lung, lysosomes were statistically significantly enlarged in AAV-GD1 mice at 4 months old compared to wild type (*p* = 0.0324) (Figure 7D), as observed previously, which was restored to wild-type levels in treated AAV-GD1 animals (*p* = 0.0261 vs. untreated AAV-GD1). In 6-month-old mice, the AAV-GD1 cohort displayed significantly increased LAMP1 staining compared to wild type (*p* = 0.0191). There was no statistically significant difference between the treated AAV-GD1 cohort and age-matched wild-type controls (*p* = 0.0844) or the untreated AAV-GD1 mice (*p* = 0.8157). However, the mean LAMP1 immunoreactivity of 6-month-old treated AAV-GD1 mice tended to be higher than that seen for age-matched wild-type controls (20.55 vs. 9.747, respectively). Light microscopy images of 4-month-old treated AAV-GD1 LAMP1-stained lungs revealed punctate lysosomal staining. This was similar to the lysosomal appearance observed in age-matched wild-type tissue (Figure 7C). In the liver, LAMP1 staining at 4 months old was similar between AAV-GD1 mice and WT controls, while treated AAV-GD1 animals displayed significantly increased LAMP1 staining (*p* = 0.0182 and *p* = 0.0192, respectively) (Figure 7F). On closer examination with light microscopy, these lysosomes did not appear enlarged, as is typical of Gaucher disease, but instead the staining appeared punctate and similar to that seen in WT liver tissue (Figure 7E). At 6 months old, both untreated and treated AAV-GD1 cohorts displayed similar average immunoreactivity values to that seen in wild-type tissue.

**Figure 8:**
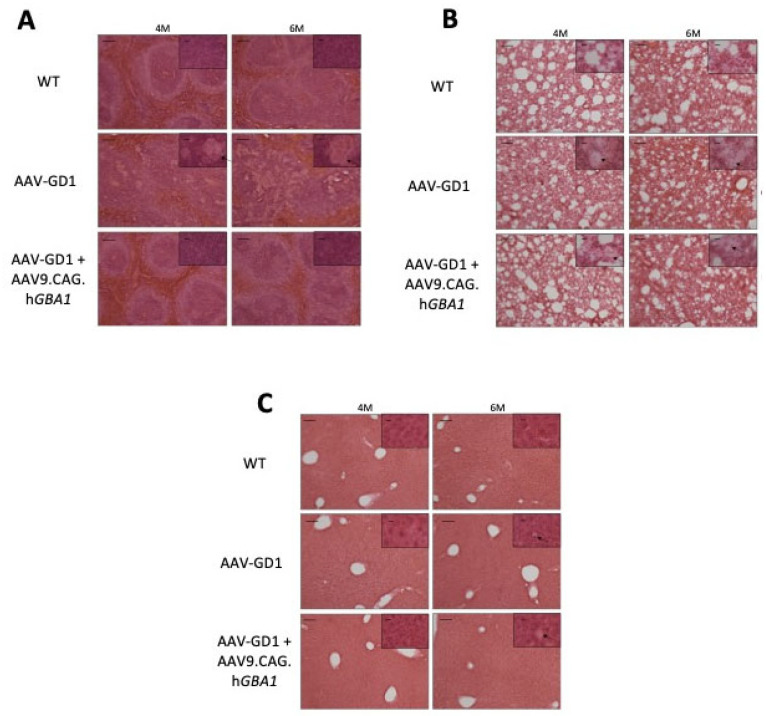
Intravenous gene therapy rescues spleen architecture in treated AAV-GD1 mice. H&E staining of (**A**) spleen, (**B**) lung, and (**C**) liver tissue from WT, AAV-GD1 mice and AAV9.CAG.h*GBA1*-treated AAV-GD1 mice revealed Gaucher cells (black arrow), and disrupted tissue architecture was rescued in treated mice, particularly in the white pulp region of the spleen at both 4 and 6 months old. Scale bar: 100 µm; higher magnification insert: 65 µm.

In untreated AAV-GD1 animals, H&E staining revealed significant disruption of spleen tissue, particularly the white pulp regions, which was rescued in treated AAV-GD1 animals (Figure 8A). In the lung, infiltrating Gaucher cells were present in both the untreated and treated AAV-GD1 mice at 4 and 6 months old (Figure 8B). H&E staining of the liver revealed no significant changes in tissue architecture at 4 or 6 months old in all cohorts (Figure 8C). At 6 months old, sporadic Gaucher cells were observed in the liver tissue of both the untreated and treated AAV-GD1 groups, but these were small, consistent with the CD68 and LAMP1 immunostaining findings.

### 3.4. Intravenous Gene Therapy Temporarily Increased β-Glucocerebrosidase Activity

**Figure 9:**
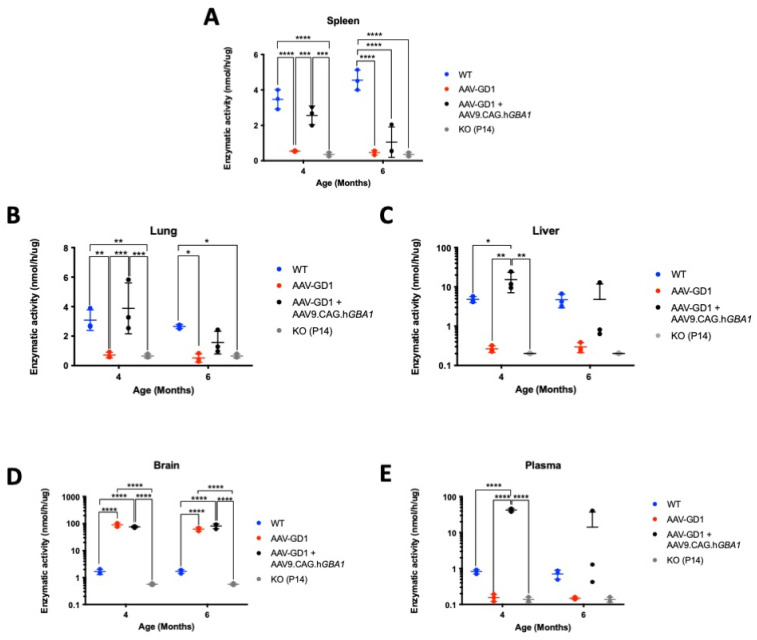
Intravenous gene therapy temporarily increases β-glucocerebrosidase activity in visceral organs. Enzymatic activity in the (**A**) spleen, (**B**) lung, (**C**) liver, (**D**) brain, and (**E**) plasma of untreated and AAV9-treated AAV-GD1 mice compared to age-matched wild-type and P14 KO controls. Initial increases in the visceral organs and plasma were observed at 4 months old, which decreased in the 6-month-old group, while enzymatic activity in the brain was maintained at supraphysiological levels. (Data presented as average ± SD. Statistical analysis: two-way ANOVA, adjusted for multiple comparisons using Sidak’s multiple comparison test. * *p* < 0.05; ** *p* < 0.01; *** *p* < 0.001; **** *p* < 0.0001).

We measured the enzymatic activity of β-glucocerebrosidase in organs and blood plasma at 4-month and 6-month timepoints. To assess the difference in enzymatic activity compared to tissue from untreated knockout animals, tissue samples from untreated K14-lnl/lnl (KO) collected at the P14 endpoint were included in the analysis as a control. In the spleen, untreated AAV-GD1 mice had significantly reduced enzymatic activity compared to wild-type and treated AAV-GD1 mice (*p* < 0.0001 and *p* = 0.0004, respectively), and it was comparable to P14 KO animals (Figure 9A). The splenic enzymatic activity in treated AAV-GD1 animals was similar to wild-type mice at 4 months old; however, this decreased in the 6-month-old cohort. At 6 months of age, both untreated and treated AAV-GD1 animals had significantly reduced enzymatic activity (*p* < 0.0001 for both groups) and were comparable to P14 KO animals. In the lung, similar results were observed to those in the spleen (Figure 9B). Four-month-old AAV-GD1 mice had significantly decreased β-glucocerebrosidase activity compared to wild-type controls (*p* = 0.0063) and treated AAV-GD1 animals (*p* = 0.0004), which was comparable to untreated KO mice. In comparison, both wild-type and treated AAV-GD1 mice showed significantly increased enzymatic activity in the lung in comparison to the untreated P14 knockout (*p* = 0.005 and *p* = 0.0003, respectively). At 6 months old, the enzymatic activity in treated AAV-GD1 animals tended to be lower than wild-type controls, while the untreated AAV-GD1 and P14 KO mice had significantly reduced enzymatic activity (*p* = 0.0143 and *p* = 0.0228, respectively). Liver enzymatic activity in 4-month-old treated AAV-GD1 animals was significantly increased compared to wild-type controls (*p* = 0.0266) and untreated AAV-GD1 mice (*p* = 0.0013), as well as in P14 KO controls (*p* = 0.0013) (Figure 9C). Untreated AAV-GD1 animals displayed comparable liver enzymatic activity to P14 KO controls at both 4 and 6 months of age. Enzymatic activity tended to decrease between 4 and 6 months in the treated AAV-GD1 mice, and no statistically significant differences were observed between any of the cohorts at 6 months of age. We also investigated enzymatic activity in the brain (Figure 9D) and plasma (Figure 9E) to assess the change in enzymatic activity afforded by the second administration of intravenous AAV9 gene therapy. In the brain, both untreated and treated AAV-GD1 mice had supraphysiological enzymatic activity at 4 (*p* < 0.0001 for both cohorts) and 6 months of age (*p* < 0.0001 for both cohorts). No changes were seen in treated AAV-GD1 animals compared to untreated, suggesting that the intravenous second injection at 2 months old does not lead to a further increase of enzymatic activity in the brains of treated mice. β-glucocerebrosidase is a soluble secreted enzyme, and therefore we examined the enzymatic activity in plasma at the sample collection point to determine the effect of a second intravenous gene therapy administration on circulating enzyme levels. At 4 months old, supraphysiological enzymatic activity levels were detected in the plasma of treated AAV-GD1 mice in comparison to wild-type and untreated AAV-GD1 cohorts, as well as P14 KO animals (*p* < 0.0001 for all cohorts). As with the visceral organs, mean enzymatic activity in the plasma of treated AAV-GD1 mice decreased between 4 and 6 months old. Additionally, there was a large amount of variation in the 6-month-old cohort, making comparison difficult. Despite this, the mean enzymatic activity in the plasma of treated 6-month-old AAV-GD1 remains increased in comparison to untreated AAV-GD1 animals and P14 KO and is comparable to wild-type enzymatic activity.

## 4. Discussion

The primary aims of this study were to investigate the following: (i) developing a novel model of type 1 GD which was more rapidly progressing compared to other models and showed the characteristic visceral pathology, and (ii) whether an intravenous AAV-mediated gene therapy approach could improve visceral pathology in this model. Here, we demonstrate the creation of a novel viable and chronic type 1 GD model, AAV-GD1, with an extended lifespan (>6 months), which develops the splenomegaly and immunohistochemical phenotypes characteristic of GD at an early stage. We also show that intravenous gene therapy administered when visceral pathology is already established can have a significant therapeutic effect.

AAV-GD1 mice were significantly smaller than age-matched wild-type controls from 4 months old, similar to results seen in the *Gba^L444P/L444P^* mouse model [34]. This may be evidence of hypermetabolism, which has been described in patients with Gaucher disease [35] which was not addressed by the AAV9.hSynI.h*GBA1* ICV localised administration. AAV-GD1 mice also developed the splenomegaly characteristic of Gaucher disease, which was evident from 2 months old. This is earlier than the splenomegaly development in the type 3 GD 4L/PS-NA model [36], GD mouse model [23], and the GBA1 model [24], which was 3 months, 12 months and 14 months, respectively. The lack of quantitative data from other studies makes comparison with the AAV-GD1 model presented here difficult. Here, we have shown statistically significantly increased macrophage activation compared to age-matched wild-type controls in the lung and spleen from 2 months old, with significant differences in the liver tissue at 4 and 6 months of age. The Gba^L444P/L444P^ [34] and point mutation models [22] did not develop splenomegaly. This early onset of splenomegaly is beneficial for future research into novel therapeutic agents for GD, allowing for shorter experimental periods and faster investigation to determine the efficacy of potential therapies.

The quantification of immunohistochemical staining to characterise the temporal development of visceral pathology in this study is unique. Further studies characterising the glycosphingolipid profile, in particular glucosylceramide and glucosylsphingosine, within organs taken from the AAV-GD1 model would provide more validation of the GD pathology. In the development and characterisation of other type 1 GD models, no quantification of visceral pathology has been reported [22,23,24,34]. Gaucher cells were found in the liver and spleen of these type 1 models at time points ranging from 12 weeks to 14 months [22,23,24,34]. Here, we have shown statistically significantly increased macrophage activation in the lung and spleen of AAV-GD1 mice from 2 months old, with significant differences in the liver tissue from 4 months of age. Although the organ systems most affected in type 1 GD patients were examined, further characterization of the haematological and osteological phenotypes of this model are required. This is necessary as anaemia, cytopenia and bone disease are common features of type 1 GD, which were not characterised in this study. Moreover, lipidomic assessment of glucosylceramide accumulation in the viscera of AAV-GD1 mice would also prove useful to assess the extent of substrate accumulation. K14-lnl/lnl mice have increased levels of glucosylceramide in the brain, liver, and spleen at birth [28], with statistically significant increases in certain isoforms observed in the brain at P12 [27]. However, to our knowledge, this novel model of type 1 GD demonstrates the earliest onset of visceral storage pathology to date.

The final aim of this study was to investigate whether an intravenous administration of AAV9.CAG.h*GBA1* to adult AAV-GD1 mice could reverse or ameliorate established visceral pathology. The inability of the gene therapy to rescue the small body weight of AAV-GD1 mice was unsurprising. This is likely due to potential disease features causing weight restriction, such as hypermetabolism [35], being too far established for effective intervention by 2 months old. However, the splenomegaly was reversed and ameliorated by the AAV9.CAG.h*GBA1* gene therapy, which was supported by reduced CD68 and LAMP1 staining results in the spleen. Post-symptomatic gene therapy can therefore reverse pre-existing storage pathology characteristic of GD. This is comparable to the rescue of splenomegaly in the GD1 mouse model [23] following transplantation of HSCs transduced ex vivo with lentiviral gene therapy in 6–9 month old mice [37]. The authors demonstrate significantly reduced splenomegaly in treated mice compared to the untreated control, 20 weeks post-HSCT administration [37]. Similar reduction in the number of Gaucher cells in the spleen following delayed treatment to other type 1 models has been previously reported [23,38,39]. However, no quantification of storage pathology was performed in these studies.

In contrast to the spleen, organ systems such as the lungs remain more refractory to treatment. The transient improvement in macrophage activation and lysosomal accumulation in 4-month-old treated AAV-GD1 mice is encouraging. However, the evidence of severe pathology in 6-month-old treated mice requires further investigation, where a reduction in enzymatic activity and consequent increase in CD68 and LAMP1 immunostaining was observed. Lung pathology in GD patients is understudied [14], and only one other study investigated efficacy of gene therapy in treating the lung, where similar results were observed following AAV8-GBA1 administration [39]. The authors suggest that the limited effect of increased glucocerebrosidase in the lung may be due to extra-lysosomal substrate in regions of the lung that have not yet been identified [39]. The potential for enzymatic supplementation with ERT in combination with gene therapy may also be a potential solution to a decline in activity following gene therapy administration.

No marked liver pathology was evident in the AAV-GD1 mice at 4 and 6 months old. It is therefore difficult to ascertain the possible effect of delayed IV administration of the AAV9.CAG.*hGBA1* vector on the liver pathology. β-glucocerebrosidase expression in the visceral organs, and particularly the liver, could be due to variation in injection administration leading to increased AAV9.hSynI.h*GBA1* vector escaping the CNS and subsequently targeting the liver, owing to the high hepatic affinity of the AAV9 vector [40]. We appreciate the group sizes in this study were small, and it is possible that larger groups would have yielded statistically significant differences particularly regarding liver pathology.

Measurements of enzymatic activity in the affected tissues two months post-AAV9.CAG.*hGBA1* administration revealed supraphysiologic levels in the liver and plasma, which is unsurprising based on the tissue tropism of AAV9 [40], and the secreted nature of glucocerebrosidase. Moreover, enzymatic activity in the spleen and lung was restored to wild-type level. A decrease in enzymatic activity was observed in all tissues, excluding the brain, when analysed from tissues four months post-AAV9.CAG.h*GBA1* administration. Similar results have been observed previously [41]. This is likely due to AAV vectors being largely non-integrating and they are instead maintained as episomes inside the nuclei of transduced cells. Cell division may therefore lead to the dilution of episomal AAV DNA, particularly in organs characterised by high cell turnover like the liver. Interestingly, previous studies looking at intravenous AAV8 gene delivery to a mouse model of type 1 GD reported a similar decrease in glucocerebrosidase from 2 to 4 months post-administration. However, this initial decrease stabilised up to 6 months post-administration [39]. Therefore, extended studies may be necessary to determine the long-term pattern of glucocerebrosidase enzymatic activity in this model and the subsequent effects on pathology development. Although we observe therapeutic efficacy to the sequential dosing of AAV9 in the AAV-GD1 model, for all the reasons above it is difficult to gauge if this would be an approach that would be feasible in the clinic. Furthermore, the first dose would require to be given during the perinatal stage which has not yet been investigated in humans. Early administration of gene therapy appears to be most effective in treating neurological aspects of the disease, however the non-integrating nature of AAV vectors means this may be less suitable for visceral manifestations. Therefore, improved delivery methods may be required to improve the longevity of visceral transgene expression resulting from AAV transduction. Recent developments in integrating AAV vectors [42] may be useful in enhancing the duration of therapeutic efficacy, with or without dual administration; however, further research would be required to determine if this is suitable to treat Gaucher disease.

One of the major issues encountered with virus-based gene therapy strategies is the development of a T-cell immune response against either the therapeutic protein, and/or humoral immunity against the viral capsid itself [43]. It is therefore important to address the possible problematic issue with a repeated administration of an AAV9 capsid serotype in this study. Reports from human gene therapy in haemophilia B patients revealed that a cellular immune response was raised against the vector capsid proteins, reducing factor IX expression [44,45,46] and could prevent repeated administration [47,48]. Reports indicate that perinatal gene transfer has shown a reduced, or absent, immune response to the transgene protein or viral capsid following AAV administration [43,49,50,51]; however, this may depend on the capsid used and organ system of interest [52]. Despite the brain being an immune-privilege organ, using the AAV-GD1 model may not be ideal for testing the efficacy of a second AAV9 vector, due to the potential of neutralising antibodies developing from the initial brain injection. With regards to further investigations using post-symptomatic AAV9.CAG.*hGBA1* intravenous administration, the potential development of an immune response against the AAV9 capsid could be eluded through serotype switching [53,54] or capsid engineering [55]. This could be achieved using other AAV serotypes or brain-targeted engineered capsids, such as AAV-PHP.B [56], AAV-PHP.eB [57], or BI-hFTR1 [58], to create a new version of the AAV-GD1 model.

Despite these limitations, the AAV-GD1 model described here could still be used to study the efficacy of other AAV gene therapies and gene delivery systems, HSCT, or small molecule therapies for type 1 Gaucher disease in a time-effective manner. The AAV9.CAG.*hGBA1* vector also has potential to reverse pre-existing Gaucher pathology, and to be used as a single gene therapy product for all forms of GD. Mice appeared healthy at the 6-month collection timepoint, and therefore future studies further extending the lifespan could be used to investigate potential alpha-synuclein pathology given the link of GBA1 mutations to Parkinson’s disease. Future work using engineered thermostable enzymes may also show greater efficacy when combined with AAV technology in the AAV-GD1 model [59,60]. While pre-existing visceral pathology is largely successfully reverted by current ERT standards of care, the attractiveness of a single administration allowing lifelong therapy cannot be understated. The future of gene therapy for Gaucher disease will largely depend on the outcomes of the clinical trials that are currently ongoing (NCT05324943).

## Data Availability

Dataset available on request from the authors.

## Abbreviations

The following abbreviations are used in this manuscript:

AAV	Adeno-associated virus
ARRIVE	Animal Research Reporting of In Vivo Experiments
AWERB	Animal Welfare and Ethical Review Board
BBB	Blood brain barrier
ERT	Enzyme replacement therapy
GCase	β-glucocerebrosidase
GD	Gaucher disease
GD1	Gaucher disease type 1
GlcCer	Glucosylceramide
GlcSph	Glucosylsphingosine
H&E	Hematoxylin and Eosin
ICV	Intracerebroventricular
IV	Intravenous
IVC	Individually ventilated cages
KO	Knockout
LSD	Lysosomal storage disease
nGD	Neuronopathic Gaucher disease
P0	Postnatal day 0
P1	Postnatal day 1
PBS	Phosphate Buffered Saline
PFA	Paraformaldehyde
SRT	Substrate reduction therapy
WT	Wild type
